# Designing a financial wellness curriculum for paediatrics residents—an educational needs assessment

**DOI:** 10.1093/pch/pxaf072

**Published:** 2025-07-31

**Authors:** Linlei Ye,, Sarah Foster,, Jessica L Foulds,, Christopher Gerdung,

**Affiliations:** Department of Pediatrics, Faculty of Medicine & Dentistry, University of Alberta, Edmonton, Alberta, Canada; Division of General and Community Pediatrics, University of Alberta, Edmonton, Alberta, Canada; Division of Pediatric Hospital Medicine, University of Alberta, Edmonton, Alberta, Canada; Division of Pediatric Respirology, University of Alberta, Edmonton, Alberta, Canada

**Keywords:** Financial literacy, Residency, Medical education, Curriculum development

## Abstract

**Objectives:**

Resident physicians face important financial decisions; however, formal education in financial literacy within residency programs is lacking. Current literature reports that residents have poor financial knowledge, increasing levels of debt, and associated anxiety. There have been no published reports on financial well-being among paediatrics residents in Canada.

**Methods:**

Using Kern’s six-step approach for curriculum development, a targeted educational needs assessment was performed. Surveys were distributed to assess residents’ financial knowledge, interest, behaviours, and to evaluate early career paediatricians’ perceived readiness for managing finances upon graduation. Descriptive statistics were performed in Microsoft Excel.

**Results:**

Seventy-eight participants (35 residents and 43 early career paediatricians) were included. Seventeen (63.0%) respondents were very interested in participating in a financial curriculum, and 18 (66.7%) respondents strongly agreed that formal education in financial literacy in residency is important.

**Conclusions:**

Participants expressed a strong desire for a financial curriculum, which represents an educational gap in residency.

Throughout training, residents face important and consequential financial decisions but are often ill-equipped with the necessary financial literacy skills (1–3). While achieving medical competence remains a cornerstone of residency training, financial literacy education is lacking (1,2). Developing skills in practice management is often not emphasized until the end of training (4). Educational seminars may be hosted by external wealth management organizations that have their own agenda and may not present information in the best interest of physicians (5). This leaves graduating residents vulnerable to misinformation and puts their fiscal health in jeopardy.

Considering the increasing levels of educational debt, pervasive deficits in financial literacy are concerning. According to the Association of Faculties of Medicine in Canada, the median debt of graduating medical students is $90,000 with 12% reporting greater than $200,000 of debt related to their medical studies (6). Low financial literacy and increased debt are correlated with higher levels of burnout, career regret, and decreased professional satisfaction (3,7,8). Moreover, Kovar et al. report that current financial wellness significantly impacts career choices and personal relationships (9). Resident physicians are susceptible to growing rates of burnout given they are earning the lowest income of their career trajectory while carrying the maximum amount of debt.

Within Canada, there is a paucity of literature evaluating financial wellness among residents, and no studies report on the financial well-being of paediatrics residents. The existing international literature unanimously reports poor financial knowledge, high debt burden, and few educational opportunities (1–3). Studies have shown that residents have a strong desire for a financial curriculum integrated into their residency program (1–3,10). This points towards a significant unmet need in medical education. Furthermore, managing finances and health resources is included as an educational objective in The Royal College of Physicians and Surgeons Paediatrics Key Enabling Competencies under Leader section 4.1.2, a blueprint that outlines important skills for paediatricians transitioning to practice (4). The national standard for recommended training experiences advocates for learning to manage a practice, including finances and human resources. This is also reflected in the transition to practice Entrustable Professional Activity (EPA) for managing longitudinal aspects of patient care in an outpatient general paediatrics setting. Under this EPA, there are specific milestones for maintaining clinic flow and managing clinic booking and scheduling, which have implications for remuneration.

Given this gap, we conducted a needs assessment to evaluate financial wellness among general paediatrics residents in our local Canadian residency program. The perspectives of early career paediatricians, who had recently graduated from our program, were included to provide further insight, directly informing subsequent curricular development.

## METHODS

### Study design

The study design is based on Kern’s six-step approach for curriculum development, with a focus on steps 1 and 2 (11). Step 1, problem identification and general needs assessment, involves defining the problem, reviewing current practices and identifying the ideal approach to designing a financial curriculum for medical residents. A core group of investigators (L.Y., C.G., JLF., S.F.) compiled existing financial education opportunities within the residency program and conducted a general needs assessment through literature review and conversation with residents.

Step 2, targeted needs assessment, was performed through administering a survey exploring residents’ financial knowledge, interests, and behaviours. Given that no tools existed with validity evidence, novel surveys were designed, piloted, and hosted in REDCap, a secure web-based database. The survey distributed to early career paediatricians included specific questions evaluating their perceived readiness for managing financial aspects of independent practice upon graduation.

Local REB approval was obtained. Participation was voluntary and implied consent was obtained with survey completion.

### Sample size

Residents enrolled in postgraduate years (PGY) 2, 3, and 4 in the University of Alberta General Pediatrics residency program during the academic year from July 1, 2022 to June 30, 2023, were included. Recent graduates from our local program from the past 4 years comprised the early career/ subspecialty participants.

Subspecialty residents who have graduated from other residency programs were excluded to allow evaluation of opportunities within the same residency program. Residents enrolled in PGY 1 were excluded as they did not have sufficient time to explore existing opportunities within the program since the survey was administered early in the academic year.

### Outcome measures

The primary objective was to conduct a financial literacy needs assessment of general paediatrics residents and early career paediatricians who have graduated from the University of Alberta paediatrics program. The secondary objectives included assessing (1) baseline knowledge of financial topics pertaining to medical practice, (2) interest in learning about different topics in finance, (3) behaviours for wealth management, and (4) identifying educational opportunities and gaps.

### Data analysis

Descriptive statistics were analysed in Microsoft Excel by a biostatistician and presented in table and graph format.

## RESULTS

A total of 78 participants, 35 residents and 43 early career paediatricians, were included. The survey response rate was 77.1% (*n* = 27) for residents and 32.6% (*n* = 14) for early career paediatricians (Table 1). Of the 41 respondents, 68.3% (*n* = 28) were female, 26.8% (*n* = 11) were male and two preferred not to answer. Most participants, 85.4% (*n* = 35), were between 25 and 35 years of age.

**Table 1.** Baseline demographics

	Current paediatrics residents	Early career paediatricians
**Total number of participants**	27	14
**Gender**		
Female	17 (63%)	11 (78.6%)
Male	9 (33.3%)	2 (14.3%)
Prefer not to answer	1 (3.7%)	1 (14.3%)
**Age**		
25–30	18 (66.7%)	1 (7.1%)
30–35	6 (22.2%)	10 (71.4%)
35–40	1 (3.7%)	1 (7.1%)
40+	1 (3.7%)	0 (0%)
Prefer not to answer	1 (3.7%)	2 (14.2%)
**Level of training**		
PGY2	5 (18.5%)	0
PGY3	8 (29.6%)	0
PGY4	11 (40.7%)	0
PGY5+	1 (3.7%)	3 (21.4%)
Early career staff (within the first 5 years of practice)	1 (3.7%)	9 (64.3%)
Prefer not to answer	1 (3.7%)	2 (14.2%)
**Number of incomes contributing to the household**		
1	13 (48.1%)	1 (7.1%)
2	13 (48.1%)	11 (78.6%)
Prefer not to answer	1 (3.7%)	2 (14.2%)
**Number of dependents**		
0	18 (66.7%)	8 (57.1%)
1	4 (14.8%)	2 (14.3%)
2	3 (11.1%)	1 (7.1%)
3	0 (0%)	0 (0%)
4	1 (3.7%)	0 (0%)
Prefer not to answer	1 (3.7%)	3 (21.4%)
**Educational debt**		
$0–$50,000	8 (29.6%)	7 (50.0%)
$50,001–$100,000	6 (22.2%)	0 (0%)
$100,001–$150,000	5 (18.5%)	1 (7.1%)
$150,001–$200,000	2 (7.4%)	0 (0%)
$200,001–$250,000	1 (3.7%)	3 (21.4%)
$250,001–$300,000	3 (11.1%)	1 (7.1%)
Greater than $300,000	1 (3.7%)	0 (0%)
Prefer not to answer	1 (3.7%)	2 (14.2%)

### Educational debt

Among residents, about half (*n* = 14, 51.9%) of the respondents reported debt under $100,000 and the other half (*n* = 13, 48.1%) had debt greater than $100,000. Five (18.5%) residents reported having debt above $200,000. Approximately half of the residents (48.1%, *n* = 13) had one income contributing to the household and the other half (48.1%, *n* = 13) had two incomes. Most residents, 66.7% (*n* = 18), did not have any dependents who relied on them for financial support. Eight residents had at least one dependent.

Half of the early career paediatricians (50.0%, *n* = 7) reported having educational debt between $0 and $50,000. Five (35.7%) respondents reported educational debt greater than $200,000. While most respondents (57.1%, *n* = 8) did not have any dependents, three (21.4%) early career paediatricians had at least one dependent.

### Residents’ self-reported financial knowledge, interest, and behaviours

Most residents (88.9%, *n* = 24) had no previous formal education in financial literacy. Participants reported the most comfort with creating a budget. Residents had the least knowledge of topics that were directly related to medical practice, including building a professional team (lawyer, accountant, etc.), licensing, and paperwork for starting practice and billing. These were the same topics that residents expressed the most interest in learning.

Seventeen (63.0%) respondents were very interested in participating in a financial wellness curriculum, and eighteen (66.7%) respondents strongly agreed that it is important for residents to have formal education in financial literacy. Greater than half of residents (55.6%) feel very or slightly uncomfortable asking preceptors about the financial aspects of practicing medicine. Eleven (40.7%) participants strongly disagree that our paediatrics residency program currently has sufficient teaching in financial wellness. Moreover, 37% (*n* = 10) of the participants agree or strongly agree that their amount of debt causes significant stress and/ or anxiety.

### Insights from early career paediatricians

Twelve (85.7%) respondents strongly disagree or disagree that they were well-equipped with the financial knowledge and skills for independent practice upon graduating from residency. A common theme that emerged was the need for transparency and specific examples to empower practical financial literacy.

## DISCUSSION

Paediatric residents at our local institution have a strong desire for formal education in financial literacy within their residency training. They expressed the greatest interest in learning about topics that they perceived to have the least comfort with. The most highly rated topics were building a professional team, licensing, and paperwork for starting practice and billing. These are topics included in our local curriculum in the final year of training. The anticipatory anxiety associated with the skills and knowledge required to manage independent practice was notable. There are many layers of complexity to physician remuneration that are often not intuitive and time-consuming to understand. It can often feel overwhelming to start independent practice focusing on providing the best patient care while also learning about the administrative components that impact the very care provided. Moreover, residents are hesitant to inquire about finances from preceptors, resulting in missed mentorship opportunities. Both residents and early career paediatricians point to the lack of accessible and reliable resources as a barrier to improving financial literacy.

It is important to recognize that academic centres are often the location where the majority of residency training takes place for many residents, though these centres may not be able to address all aspects of financial wellness, including topics such as setting up a community practice and in some situations, billing. Physicians who typically work in the community, however, may not be as well-versed in contract negotiations and benefit packages offered by academic centres. Ensuring that residents are presented with broad information from numerous sources, including varied work practices, is an important aspect of financial wellness education. This emphasizes the need for formal teaching by both academic and community physicians.

Enhancing financial literacy education for residents is also important for improving physical well-being. A significant percentage of residents in our study experience stress and/ or anxiety associated with educational debt. Notably, 69.2% of residents carrying education debt above $100,000 reported they are very interested in participating in a financial wellness curriculum, whereas 75% of residents with debt less than $50,000 reported they were only somewhat interested. This suggests that increased debt burden may be correlated with a stronger desire to learn about financial management. Several studies have demonstrated that participation in a finance curriculum was associated with improved resident wellness and decreased financial stress (8,12–14). This impact on physician wellness highlights the importance of this not just being part of a transition to practice curriculum but a more intentional component from early on in training. Increasing transparency through formal education may help alleviate the stress associated with seeking advice from supervising preceptors and searching for resources. Coincidental informal mentorship alone is inequitable.

These results will be used to design and implement a longitudinal financial wellness curriculum grounded in steps 3 to 6 of Kern’s approach to curriculum development, which will be instituted earlier in residency (11).

Our study has several limitations. Firstly, this needs assessment was conducted in a single centre with a specific residency group and therefore, generalizability is limited. Compensation and cost of living vary geographically and influence financial planning and lifestyle considerations. Secondly, the response rate for early career paediatricians was low and therefore may not be representative. Several factors may have contributed to this, including managing competing demands as a busy paediatrician and transitioning to a new email address at a different institution or community. Thirdly, our survey did not differentiate between residents from different years of training. Therefore, it is not known if financial wellness becomes more important later in residency when trainees are preparing for transition to practice. Nevertheless, the study points to a clear educational gap for which there is notable interest. The final, not insignificant limitation is space within a packed curriculum. Time is a limited resource so how best to address this in larger curriculum mapping is essential in our local next steps.

At our local institution, we intend to implement two financial wellness seminars per postgraduate year, for a total of eight seminars, into the academic half-day curriculum. The topics are based on survey results and will be delivered sequentially to facilitate building foundational knowledge before acquiring advanced skills. The chosen topics, in order, are: introduction to financial literacy, budgeting, debt management and repayment strategies, insurance, tax planning and investment basics, understanding physician remuneration, transition to independent practice, and building a professional financial team. These seminars will be delivered by paediatricians who work in a variety of settings, with varied remuneration, to provide different perspectives. We aim to collect iterative feedback to continuously improve this curriculum.

Given the current economic milieu with rising cost of living, compounding educational debt, and delayed earning potential, there is no better time than now to prepare residents for their financial futures.

## CONCLUSION

Our study is the first to report on financial well-being among paediatrics residents in Canada. This needs assessment demonstrates a strong interest in participating in a financial wellness curriculum and current unmet needs. As we develop our longitudinal financial wellness curriculum, we call on our colleagues at other centres to consider physician finance and educational initiatives in their local contexts in addition to what is collaboratively done as part of the national transition to practice curricula.
